# Supplementary material for: Microgravity Modifies the Phenotype of Fibroblast and Promotes Remodeling of the Fibroblast–Keratinocyte Interaction in a 3D Co-Culture Model
Source: Int J Mol Sci. 2022 Feb 16;23(4):2163. doi: 10.3390/ijms23042163 (PMC8879576; doi:10.3390/ijms23042163)
Supplement: Supplementary file 1 [file ijms-23-02163-s001.zip › ijms-1555302-Supplementary.pdf]

Supplemental Material

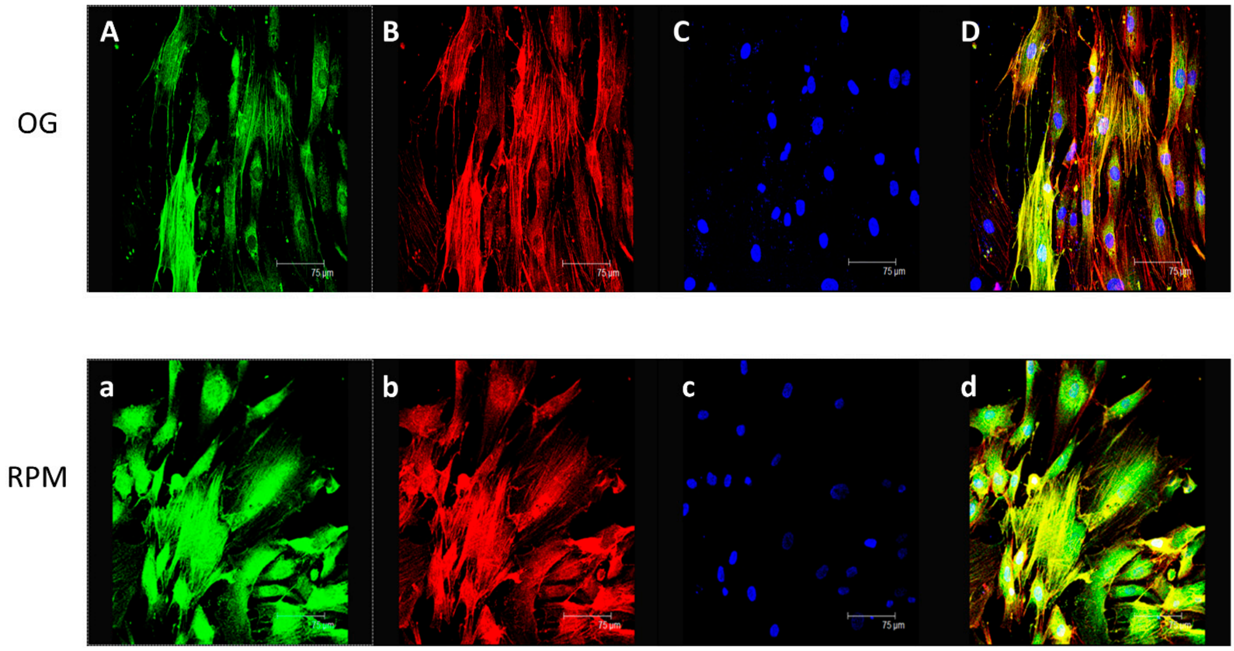

Figure S1. Representative images of colocalization analysis of α-SMA (green) and F-actin (red) in fibroblasts cultured in OG and RPM for 48 hours. It is interesting to note how the fibroblasts after 48h of exposure to microgravity adapt and the α-SMA is distributed as in the control condition. α-SMA is not present in the nucleus of fibroblasts in RPM but co-localizes with the F-actin fibers along the cell membrane (Scale bars 75 μm).

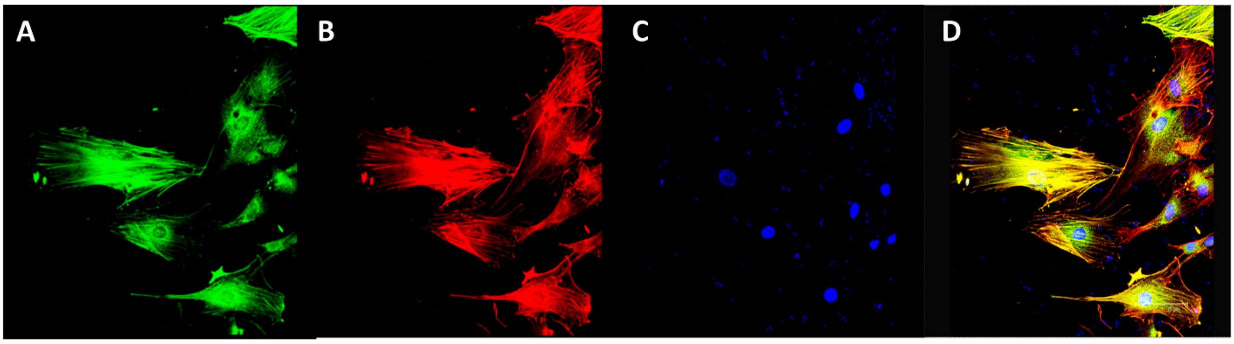

Figure S2. Representative images of colocalization analysis of α-SMA (green) and F-actin (red) in fibroblasts cultured in RPM for 24 hours and then transferred into a normal gravitational field for further 24 hours (Recovery condition). (**A**): α-SMA distribution is shown; α-SMA recovers almost completely the distribution pattern observed in *1g* cultured cells. (**B**): F-actin organization; (**C**): colocalization analysis of F-actin and α-SMA, the colocalization (green and red bars) is shown. Colocalization in recovery condition appears as observed in OG condition.

RECOVERY

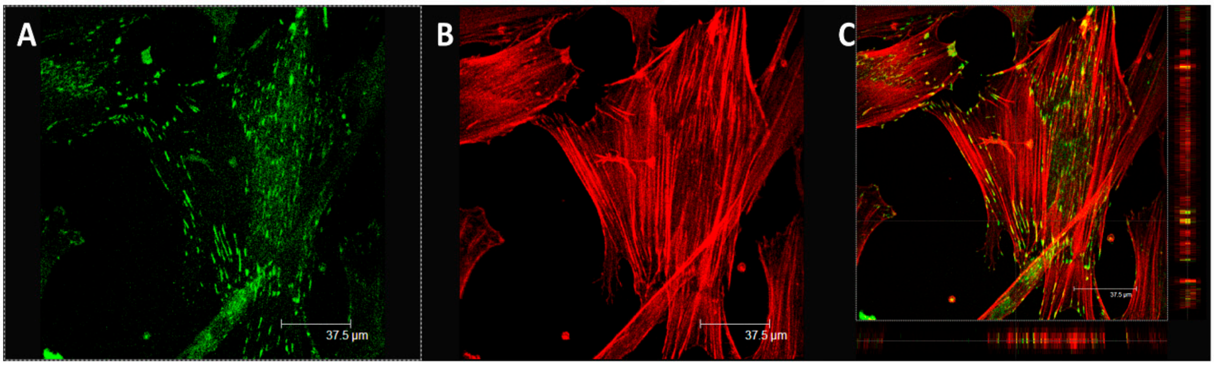

Figure S3. Representative images of colocalization analysis of vinculin (green) and F-actin (red) in fibroblasts cultured in RPM for 24 hours, then transferred into a normal gravitational field for further 24 hours (Recovery condition). (**A**): vinculin distribution is shown; vinculin recovers almost completely the distribution pattern observed in *1g* cultured cells. (**B**): F-actin organization; (**C**): colocalization analysis of F-actin and vinculin., the colocalization (green and red bars) is shown. Colocalization in recovery condition appears in focal contacts as observed in OG condition. Scale bars 37,5 µm.
